# Mitigating heat-related mortality risk in Shanghai, China: system dynamics modeling simulations

**DOI:** 10.1007/s10653-020-00556-9

**Published:** 2020-04-29

**Authors:** Xiaochen Liu, Zhan Tian, Laixiang Sun, Junguo Liu, Wei Wu, Hanqing Xu, Landong Sun, Chunfang Wang

**Affiliations:** 1 Shanghai Climate Center, Shanghai Meteorological Services, Shanghai, 200030 China; 2 Key Laboratory of Cities’ Mitigation and Adaptation to Climate Change in Shanghai, Shanghai, 200092 China; 3 School of Environmental Science and Engineering, Southern University of Science and Technology, Shenzhen, 518055 China; 4 Department of Geographical Sciences, University of Maryland, College Park, MD 20742 USA; 5 School of Finance and Management, SOAS University of London, Russell Square, London, WC1H 0XG UK; 6 International Institute for Applied Systems Analysis (IIASA), 2361 Laxenburg, Austria; 7 Key Laboratory of Geographic Information Science (Ministry of Education), East China Normal University, Shanghai, 200241 China; 8 Shanghai Center of Disease Prevention and Control, Shanghai, 200336 China

**Keywords:** Heat waves, Mortality, Public health, System dynamics model

## Abstract

Numerous studies in epidemiology, meteorology, and climate change research have demonstrated a significant association between abnormal ambient temperature and mortality. However, there is a shortage of research attention to a systematic assessment of potential mitigation measures which could effectively reduce the heat-related morbidity and mortality risks. This study first illustrates a conceptualization of a systems analysis version of urban framework for climate service (UFCS). It then constructs a system dynamics (SD) model for the UFCS and employs this model to quantify the impacts of heat waves on public health system in Shanghai and to evaluate the performances of two mitigation measures in the context of a real heat wave event in July 2013 in the city. Simulation results show that in comparison with the baseline without mitigation measures, if the hospital system could prepare 20% of beds available for emergency response to heat waves once receiving the warning in advance, the number of daily deaths could be reduced by 40–60 (15.8–19.5%) on the 2 days of day 7 and day 8; if increasing the minimum living allowance of 790 RMB/month in 2013 by 20%, the number of daily deaths could be reduced by 50–70 (17.7–21.9%) on the 2 days of day 8 and day 12. This tool can help policy makers systematically evaluate adaptation and mitigation options based on performance assessment, thus strengthening urban resilience to changing climate.

## Introduction

The fifth assessment report of the IPCC clearly stated that the world had warmed by approximately 0.85 °C from 1880 to 2012 and that extreme temperatures such as heat waves have become more frequent, intense, and long-lived (IPCC 2014). These changes will lead to an increase in heat-related deaths and bring a huge challenge to public health system.

In 2003, the summer heat wave swept over India, Eastern China, and Western European countries successively resulted in a death of over 25,000 in the Western European. France experienced the worst hit, with 14,802 deaths during the 20-day heat wave (D'Ippoliti et al. 2010). The death toll in Portugal, Spain, Italy, and even Switzerland increased by 40%, 8%, 15%, and 7% (Kovats and Haiat 2008). In 2009, Southeastern Australia suffered a heat wave which caused 374 excess deaths in Victoria during the week of January 26 to February 1 (Ren et al. 2014). In Shanghai, the heat wave of July 2013 had a significant impact on mortality even after considering “mortality displacement.” For example, the estimation in Sun et al. (2014) put the number of excess deaths at about 167 in all-cause mortality. It is well acknowledged that exposure to extreme heat wave increases mortality (Huang et al. 2012a, b; Ren et al. 2014).

A large number of studies have extensively assessed the climate impact on human health with an focus on the relationships between climate variables such as heat waves and mortality by ages, gender, disease, or economic status (e.g., among others, Semenza et al. 1999; Tan et al. 2004 ; Johnson et al. 2005; Gosling et al. 2009; Bustinza et al. 2013; Berko et al. 2014; Zheng et al. 2014; Huang et al. 2015; USGCRP 2016; Zeng et al. 2016). Some studies focus on establishing statistical relationships between abnormal ambient temperature and mortality or morbidity using regression models of time-series data (Braga et al. 2002; Curriero et al. 2002; Zeger et al. 2006). Using the absolute measure of years of life lost (YLL, Health and Social Care Information Centre of NHS 2015), which reveals the relative importance of different causes of premature death within a particular population and is calculated by summing over ages 1–74 years the number of deaths at each age multiplied by the number of years of life remaining up to age 75 years, Huang et al. (2012a, b) examined the effects of temperature on the YLL due to cardiovascular disease (CVD) in Brisbane, Australia. Their estimation based on a distributed lag nonlinear model showed that ambient temperature is associated with YLL due to CVD and that prolonged extreme heat events significantly amplify the adverse effects on YLL. Li et al. (2018) employed both general linear model and distributed lag nonlinear model to assess the nonlinear and delayed effects of temperature on the YLL of non-accident mortality in Tianjin, China, and then use the established relationship to calculate the YLL changes attributable to future climate scenarios in 2055 and 2090. They found that future climate change will worsen the temperature-related YLL. Other publications reported that difference in the socioeconomic factors, such as availability of air condition, standards of living, and housing quality, contribute to differences in hot weather-related mortality (e.g., Chesnut et al. 1998; Kovats and Hajat 2008; Mavrogianni et al. 2009). Many studies targeted at high-risk groups to identify those who were the most vulnerable to dying in a heat wave (e.g., Hajat and Kosatsky 2010; Li et al. 2018). The study of 50 US cities in Medina-Ramon and Schwartz (2007), which employed city-specific conditional logistic regression models in combination with a random-effects meta-analysis, found that larger heat effects were associated with higher population density. This finding may reflect that the urbanized settlements in megacities may experience higher temperatures due to compacted buildings, poorer ventilation, and localized heat sources. The work of Hajat and Kosatsky (2010) showed that lower GDP of a city was associated with higher heat risk because of less access to home air-conditioning and other technological protection measures. Some studies have also reported the positive role of air-conditioning in reducing heat risk (Braga et al 2001; O’Neil et al. 2005). It is also reported that vulnerability to heat is higher for babies, children, and the old because of changes in the thermoregulatory system (Grundy 2006; Empereur-Bissonnet et al. 2006).

In recent years, mitigating the impact of heat wave through urban greening and cool surfaces has attracted increasing research interests. The case study of the city of Melbourne, Australia, in Chen et al. (2014) suggests that around 5–28% and 37–99% reduction in heat-related mortality rate could be achieved by doubling the city’s vegetation coverage and transforming the city into parklands, respectively. Macintyre and Heaviside (2019) showed that cool roofs may offset heat-related mortality by mitigating the urban heat island effect during a heat wave.

Heat wave is typically defined by heat index, also known as the apparent temperature, or the ambient temperature exceeding predefined thresholds over a number of days (USGCRP 2016; Lippmann et al. 2013). However, different definitions were used in the previous studies because the impact of heat wave depends on many factors including climate, socio-demographic characteristics, and acclimatization of the population (Tong et al. 2015). The Royal Netherlands Meteorological Research Institute considers the heat wave to be a weather process in which the highest temperature is above 25 °C for more than 5 days (at least 3 days above 30 °C) (Huynen et al. 2001). By contrast, the China Meteorological Administration (CMA) stipulates that a high-temperature process with daily maximum temperature exceeding 35 °C for 5 days or exceeding 38 °C for 2 consecutive days is regarded as a strong heat wave; and a high-temperature process with daily maximum temperature exceeding 35 °C for 8 days or exceeding 38 °C for 3 consecutive days is regarded as an extreme heat wave (Tan and Huang 2004). This study adopts the definition of CMA.

From the above discussion, we can see that extensive studies have been done on the relationship between temperature and mortality or morbidity, and on investigating the determinants of heat-related mortality and morbidity and mitigation effects on them. Nevertheless, there has been a shortage of research attention to a systematic assessment of potential mitigation measures which could effectively reduce the heat-related morbidity and mortality risks (Boeckmann and Rohn 2014; Arbuthnott and Hajat 2017). In this study, we argue that a city is a complex system of sub-systems which has become increasingly vulnerable to extreme weather and climate events. A climate shock to any one of the subsystems may bring significant risk to the entire urban system. Therefore, it is necessary to study the impact of climate change on the public health system through a system thinking way. Guided by this thought, we first illustrate a conceptualization of a systems analysis version of urban framework for climate service (UFCS). We then develop a system dynamics (SD) model for the UFCS, which include five subsystems of climate, health, social, economic, and governance. Finally, we employ this SD model to quantify and assess the impacts of heat waves on public health system in Shanghai and to evaluate the performances of a short-term and a long-term mitigation measures for the system.

## Data and methods

### Data

**Fig. 1:**
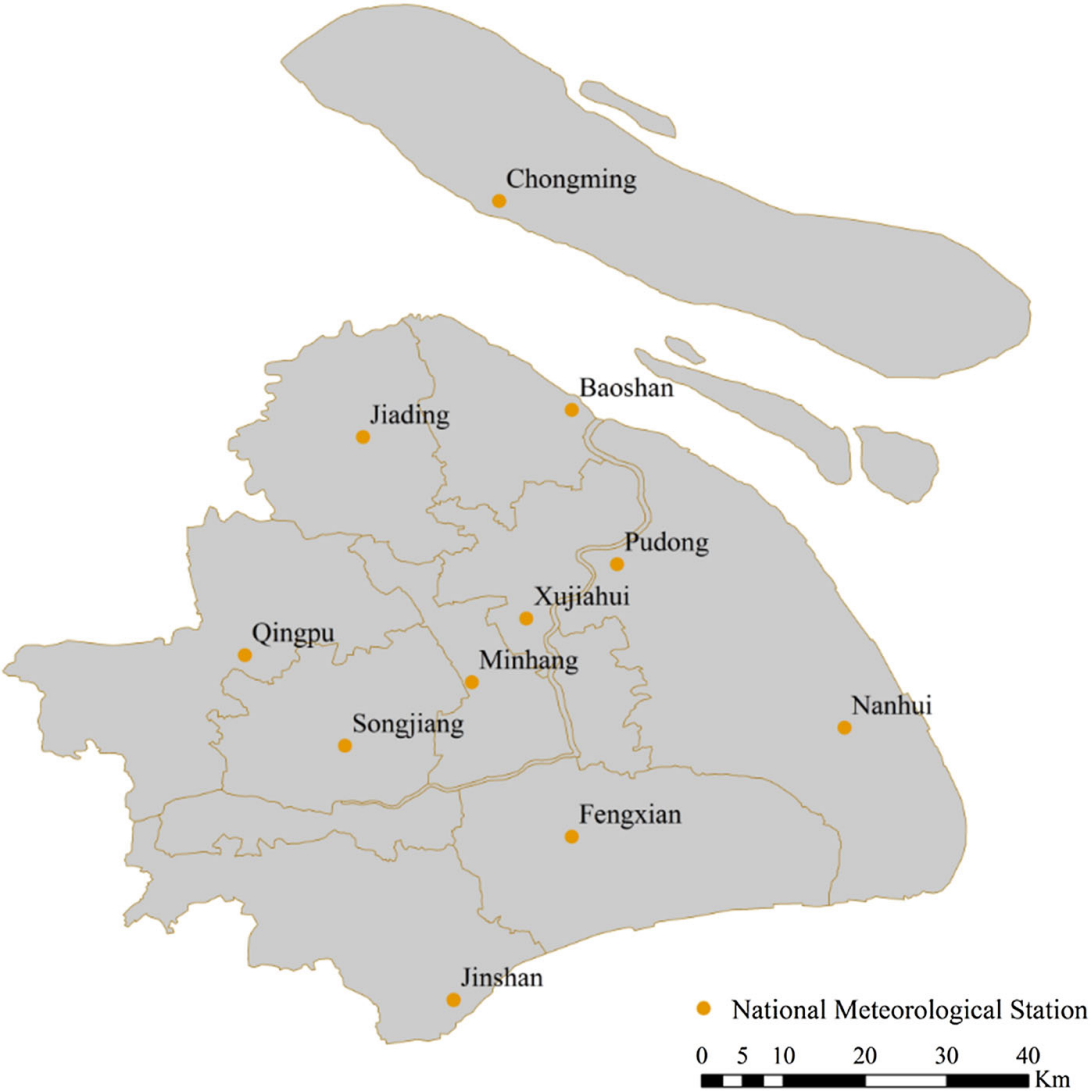
The locations of the 11 meteorological observation stations in Shanghai

Daily meteorological data between 2003 and 2013, which were obtained from Shanghai Climate Center, are the observation records of 11 Meteorological Observation stations, as shown in Fig. 1, which are located in the 11 districts of Shanghai. We took June 1 to September 30 in period from 2003 to 2013 as illustration period to match with the available data for public health.

The daily data on non-accidental deaths for Shanghai were from Shanghai Municipal Center for Disease Prevention and Control (CDC). Daily deaths data from June 1 to September 30 during the period from 2008 to 2013 were used to develop the relationships between temperature and mortality and calibrate the model. Besides that, the daily deaths data for the summer (June–September) 2003 were applied to validate the SD model.

Other social statistic data, e.g., population, personal income, and air-conditioning using rate, for period from 2003 to 2013, are from statistical yearbooks of Shanghai obtained from the Shanghai Bureau of Statistics.

### The urban framework for climate service: conceptualization

We think that the systems analysis-based approach is more capable of capturing the broad socioeconomic impacts of adverse climate events across the “system of systems” in a modern city, where human and physical systems work together to make the smooth functioning of the city. Existing climate service approaches mostly deal with individual sub-systems. In contrast, this paper promotes a quantitative approach to model the interdependencies between different systems at different scales, with a due consideration on the governing structures that influence the way systems work, which is the conceptualization of a framework for climate service, the UFCS for short.

**Fig. 2:**
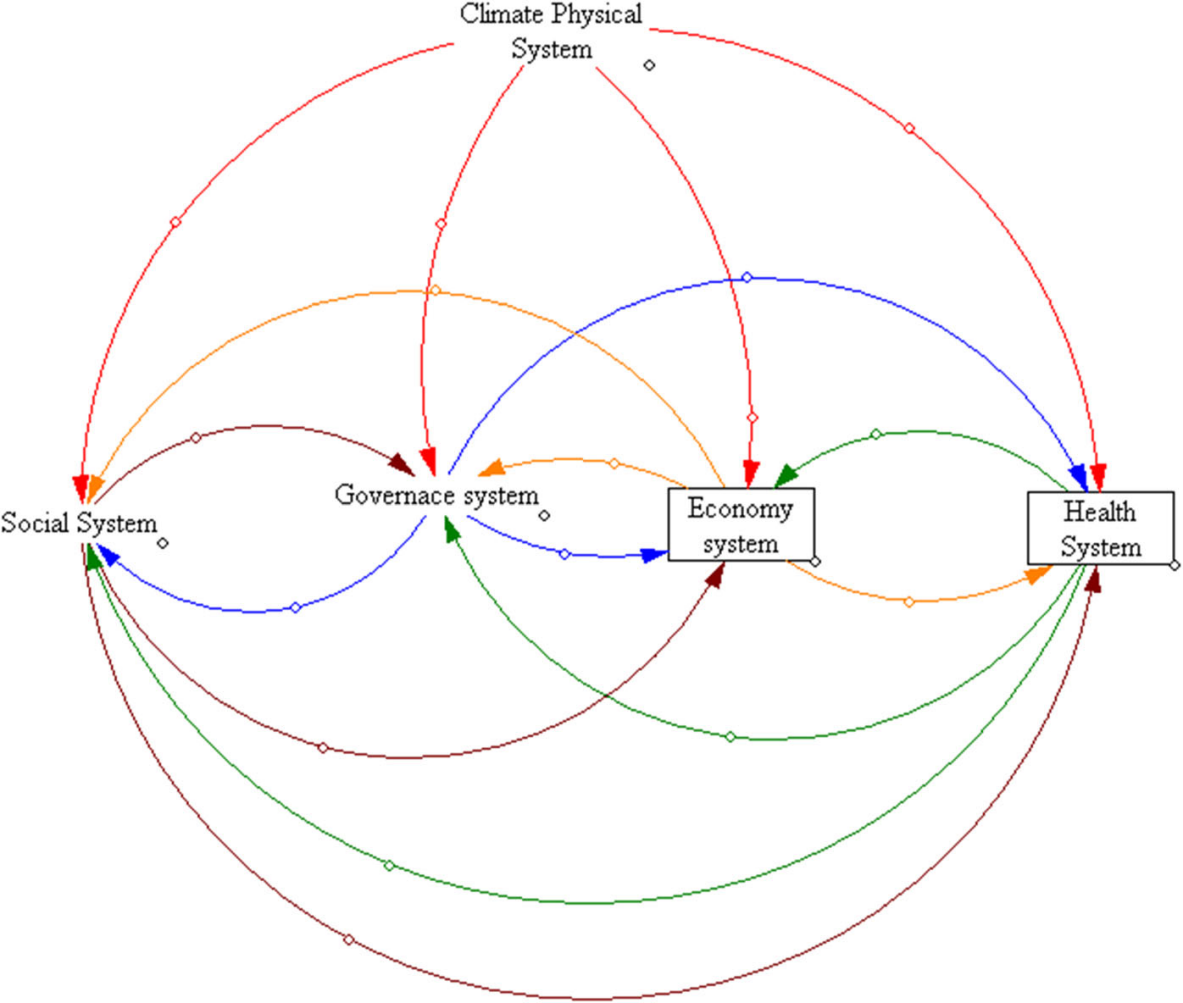
Interactive loop across the five urban systems in the urban framework for climate service (UFCS)

Climate services serve the goals of the society in terms of (a) minimizing human suffering and economic losses triggered by adverse climate events; (b) maximizing the ability and capacity of the society to adapt and adjust following climate shocks to the urban systems; (c) minimizing the cost and time span of recovery. Such minimization and maximization processes involve five important urban systems, which are climate physical system, health system, social system, economy system, and governance system. The interaction across these five urban systems can be calibrated into a dynamic loop structure using a system dynamic modeling approach (Fig. 2). The more detailed and specific setting of the loop structure will depend on the features of the triggering climate event and the socioeconomic and governance structure of the city.

### The index system for urban climate service in the case of heat wave in Shanghai

An index system which can effectively serve the above goals (a)–(c) of the urban society needs to capture the key characteristics of these five urban systems. Table 1 presents an index system of UFCS in the case of a heat event in Shanghai.

**Table 1:** Index system of UFCS in the case of a heat event in Shanghai

System	Indicator	Data sources
Climate physical system	Daily average temperature	Observation and projection
	Daily maximum temperature	
	Daily minimum temperature	
	Daily humidity	
	Hot days	
Health system	Number of medical staffs	Shanghai Statistical Yearbook
	Number of hospital	
	Number of hospital beds	
	Death toll	Shanghai CDC
Economy system	Personal income	Shanghai Statistical Yearbook (2003–2008)
	Air-conditioning using rate	
social system	Population over 65	
	Population under 14	
	Population 15–64	
Governance system	Minimum living standard	
	Social insurance coverage	

#### The climate system indicators (input indicators)

Shanghai is exposed to multiple types of climate hazards including heat wave, typhoon, storm surge, heavy rainfall, sea-level rise, and upstream flooding. These hazards drive the physical sector of the UFCS and represent the natural climate system. Climate physical system is connected to other systems in the UFCS and interacts with the whole city system. As we discussed in the introduction section, increasing research attentions have been attracted to assessing the impact of climate change on human health because the number of deaths caused by heat waves has dramatically risen in recent years.

As an international metropolitan, Shanghai has a huge population of 24.1 million with a high density of 3814 people/km^2^ and its population of over 60-year age reaches 20% of the total population in 2017 (Shanghai Statistics Bureau 2008). Under the foreseeing climate change, the high population density and aging population are going to aggravate the impact of heat wave on public health and to bring additional challenges to public health security. This justifies the focus of this study on the human health concerns caused by an extreme heat wave event in Shanghai.

Taking the case of a heat wave event as an example, it first triggers the extreme events in the climate system and then the consequent adverse impact across the whole system, Panel 1 in Table 1 presents the five important indicators which are the important climatic variables related to a heat wave event.

#### The health system indicators (system indicators)

Climate-related natural hazards can have significant health impacts on a population. In the case of a heat wave, it may trigger outbreaks of heatstroke and other diseases, which may immediately impede mobility. It is important to estimate and anticipate potential health impacts to better prepare for, respond to, and recover from a shock triggered by a heat wave to human health and socioeconomic sectors. Panel 2 in Table 1 presents the five most relevant indicators for the system dynamic modeling of the connection between the climate system and health system in the case of a heat wave shock, with data available from the Shanghai Statistics Yearbooks.

#### The economic system indicators (system indicators)

Economic system is itself complex, consisting of many inter-related sectors and exhibiting nonlinear behavior in both information and material flows. The two sets of indicators presented for this system in Panel 3 of Table 1 are the important relevant factors which influence the heat-related mortality risk, including low income (Kaiser et al. 2001) and lower access to air-conditioning (O’Neill et al. 2005). Other factors influencing heat-related mortality, such as poor housing, are not count in since the data are not accessible.

#### The social system indicators (system indicators)

The relationship between urban development, environmental degradation, and hazard vulnerability often manifests itself as a vicious, mutually reinforcing system of feedbacks especially prevalent in developing countries. The potential impacts of climate disasters on social systems may be very severe. The indicators considered in our demonstrative case of a heat wave shock are presented in Panel 4 of Table 1. Our literature review shows that elderly population is particularly and strongly affected by heat waves (e.g., Basu and Ostro 2008; Vaneckova et al. 2008). Therefore, we choose the populations by different age group as the major indicators for social factors.

#### The governance system indicators (system indicators)

The effectiveness of climate change adaptation measures must consider the political administrative and institutional framework which affects the functioning of the coastal/delta cities. This institutional framework defines the overall effectiveness of decision making. It must be framed because the overall implementation and effectiveness of climate change adaptation options depends on political motivation, budgets and climate change policy. In the case of a heat wave shock in Shanghai, we do not expect a change in governance structure. However, policy parameters such as government support, minimum living standard, and coverage of social insurance (Panel 5 in Table 1) are important for assessing the impact of the shock and recovery from the shock. It is because the degree to which people may experience the hazard damages of a heat wave event is influenced by their tolerance level and coping capabilities in stressful situations. The poorer communities are more vulnerable to hazard. The poor citizens are reluctant to use the air-conditioning to cool the house because of cost considerations. They might be reluctant to visit a hospital since they have less or none insurance. Therefore, improving the minimum living standard and increasing insurance cover rates by policy efforts could reduce the size of vulnerable population and thus reduce the mortality risk caused by the extreme heat wave event.

### Development of the system dynamic (SD) model for a heat wave shock

SD, which was developed in the 1950s by Jay Forrester at the Massachusetts Institute of Technology, is a simulation modeling method used for representing the structure of complex systems and understanding their behavior overtime (Marshall et al. 2015). The core elements of SD are feedback, accumulations (stocks), rates (flows), and time delays. Stocks are accumulations or aggregations of something (e.g., people), shown as boxes and represent the state variables or variables which increase or decrease in value over time and whose value can only be changed by flows. Flows are represented as rates over time which changes the value of a stock; these feed in and out of stocks and have the same units of stocks per time unit (e.g., hospital beds per year). Each auxiliary variable in the model represents either an equation that is a function of the inputs or a constant. Delay may be added which represents time lags to variable changes. An important concept in SD is nonlinearity (Marshall et al. 2015). In general terms, SD can produce patterns and trends, as well as mean values as outputs from the model. The patterns and trends resulting from simulation experimentation with different policies or strategies (“what-if” questions) can be analyzed by modelers and stakeholders to inform decision making.

This SD model is developed to analyze climate impact on the urban public health system and how the urban public health system response to extreme climate events, to evaluate adaptation plans, and to provide the preferred solutions. Based on the previous studies, the indexes that relate to urban system and affect mortality are mainly from three aspects: natural hazard, exposure, and vulnerability. Through systematic analysis, the climatic indexes we chose are maximum temperature and heat wave duration. The choice of maximum temperature is a result of a statistical analysis which shows that the maximum temperature and heat wave duration are the most sensitivity climatic variables on mortality compared with other factors, such as daily average temperature, minimum temperature, and humidity. Exposure factors depend on population structure and economic income level. Vulnerability factors depend on equipment level and human resource level of medical services (Table 1).

**Fig. 3:**
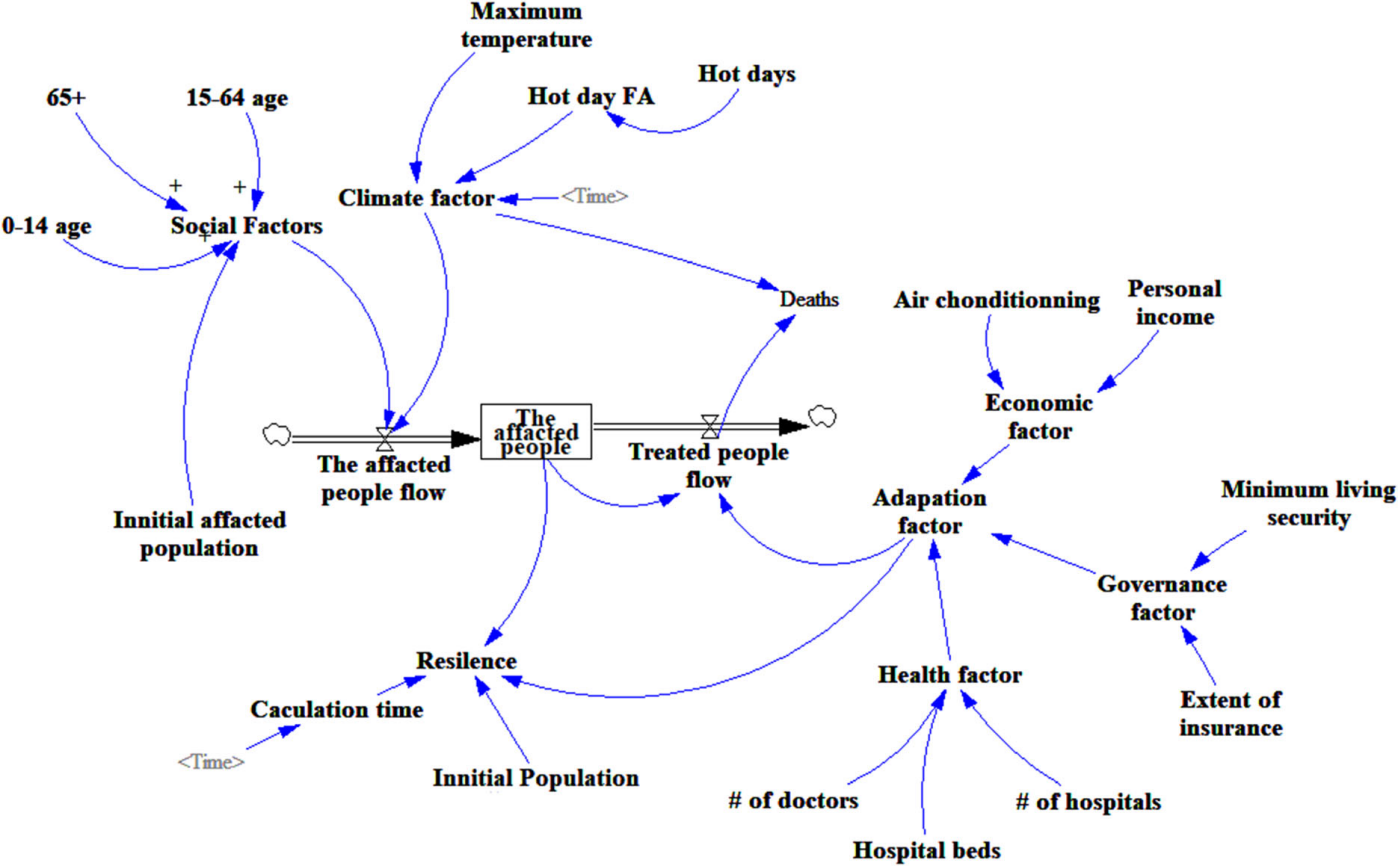
Interactive dynamics of the UFCS-SD model in the case of a heat wave shock in Shanghai

The climate system sector, as shown in Fig. 3, represents the status of the climate factors in the system related to human health. The maximum temperature, which is a table function variable in this system, represents the severity of heat wave. The number of hot days is determined by how many days that a heat wave continued.

The key stock in this system is the affected people, which is increased by the affected people flow and decreased by treated people outflows. The affected people flow is a daily input to the model, and its values are calculated based on the historical relationship between abnormal temperature and death toll records. The initial of the affected people is set by the users as a model input and its value is based on the historical records. The treated people represent people who get treated and recovered from this heat wave event.

Other initial variables are such as population by age cohort before the shock, number of hospitals, number of hospital beds, number of hospital staffs, insurance coverage, minimum living allowance, etc.

Although the system dynamics flow diagram (Fig. 3) can describe the cause–effect relationship and system structure between the various elements of the system, it cannot explicitly show the mathematic relationships between the variables of the system. There are a few but important sensitive parameters in the SD model. Minor changes in these parameters may cause significant changes in the behavior of the system. Therefore, model equations and parameter estimation are very important for quantitative analysis of system dynamics models. The functions on the major path of affected people flow, affected people, treated people flow, and death are presented as follows.

The dynamic function of affected population (AP) is specified in Eq. (1):

1 $$\frac{{\Delta {\text{AP}}_{t} }}{\Delta t} = \frac{{{\text{AP}}_{t + \Delta t} - {\text{AP}}_{t} }}{\Delta t} = {\text{APF}}_{t} - {\text{TPF}}_{t} ,$$

where AP_*t*_ is the affected population at time *t*, APF_*t*_ is the increase in the affected population (called affected people flow) between *t* and $t + \Delta t$, TPF_*t*_ is the increase in the treated population (called treated people flow) between *t* and $t + \Delta t$, and $\Delta t$ is the time step.

The central component of APF is the empirical relationship between daily maximum temperature (*T*_max_) and excessive morbidity. Zhan (2018, Chapter 2) employed daily morbidity and mortality data of Ningbo and Shanghai during June–August in 2011–2013 to establish this relationship for both morbidity (when *T*_max_ ≥ 36.6 °C) and mortality (when *T*_max_ ≥ 37.6 °C). We adopt these two relationships in our research.

TPF can be calculated using Eqs. (2)–(4).

2 $${\text{TPF}} = {\text{AP}} \cdot {\text{AF}},$$

where AF is the adaptation factors.

The climate factor (CF) is a function of maximum temperature (MA) and hot day AF (HDFA).

3 $${\text{CF}} = {\text{MA}}\left( {\Delta t} \right) \cdot {\text{HDAF}}.$$

For a number of sensitive parameters such as daily climatic variables in the model, it is difficult to show their changes just using simply equations. Therefore, table functions are employed to illustrate their trends.

The adaptation factor (AF) is calculated using Eq. (4):

4 $${\text{AF}} = {\text{EC}} \cdot {\text{HF}} \cdot {\text{GF}},$$

where EC is the economic factor determined by air-conditioning using rate and personal income, HF is the health factor determined by the number of doctors, hospital beds and number of hospitals, and GF is the governance factor represented by the minimum living allowance and extent of health insurance coverage.

The initial values in the modeling process are mostly taken from the 2004 Statistical Yearbooks of Shanghai in different areas and the 2014 Statistical Yearbooks of Shanghai, with the exception of daily death toll.

## Results

### The relationship between climate variables and mortality

**Fig. 4:**
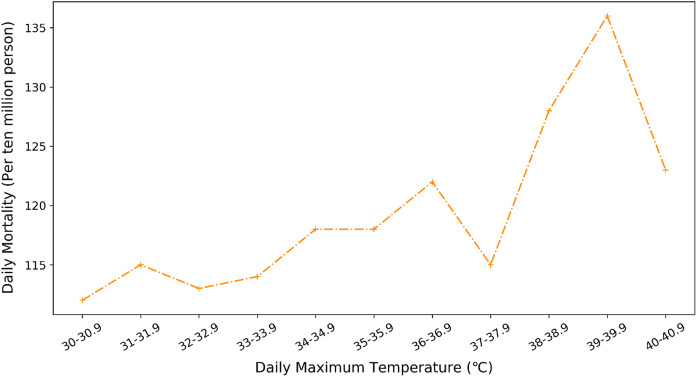
Relationship between daily maximum temperature and average daily death toll

As shown in Fig. 4, the average daily mortality in our database (2008–2013) is about 114 persons/ten million/day when the maximum temperature is under 35 °C. When the maximum temperature is over 35 °C, the average daily death toll increases to 124 persons/ten million/day with an increment of 9%. It may suggest that the summer death toll is significantly influenced by the daily maximum temperature.

### Model calibration and validation

The calibration of the model is to test the extent to which the model simulation deviates from the consolidated real system data, to check whether the information and behavior simulated by the model show the characteristics and changes of the actual system, and to consider whether we can understand and solve the problems through model simulation analysis. Our calibration was implemented based on daily weather and daily death data for summers (June 1 to September 30) in the period from 2008 to 2013, which was obtained from Shanghai Municipal Center for Disease Control and Prevention. The calibration process aimed to minimize the errors between the simulation results and the actual observations, which consists of various fitness tests of the system’s behavior with reference to the historical data. After taking many rounds of modifications and fittings, the results of the simulation loops become stable and effectiveness of the model become satisfactory.

**Fig. 5:**
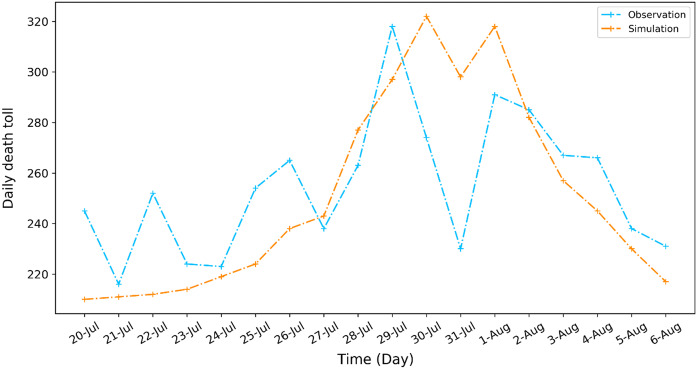
Comparison of the simulated and the observed daily death tolls during the extreme heat wave in July 2003

To validate the calibrated SD model, we use it to “predict” the impact of the heat wave on urban system from July 20 to August 6, 2003, which was the longest extreme heat wave between 1960 and 2013. The simulation is carried out for the heat wave duration of 19 days with time step of 1 day. Figure 5 compares the observed and simulated daily deaths during the 19-day of extreme heat wave. The comparison shows that the simulation results of our SD model are capable of correctly “predicting” the peaking process (with a time lag of 1 day) and peaking value of the daily death toll associated with the heat wave.

### Marginal impact of 1 °C increase on daily death toll in July 2013

**Fig. 6:**
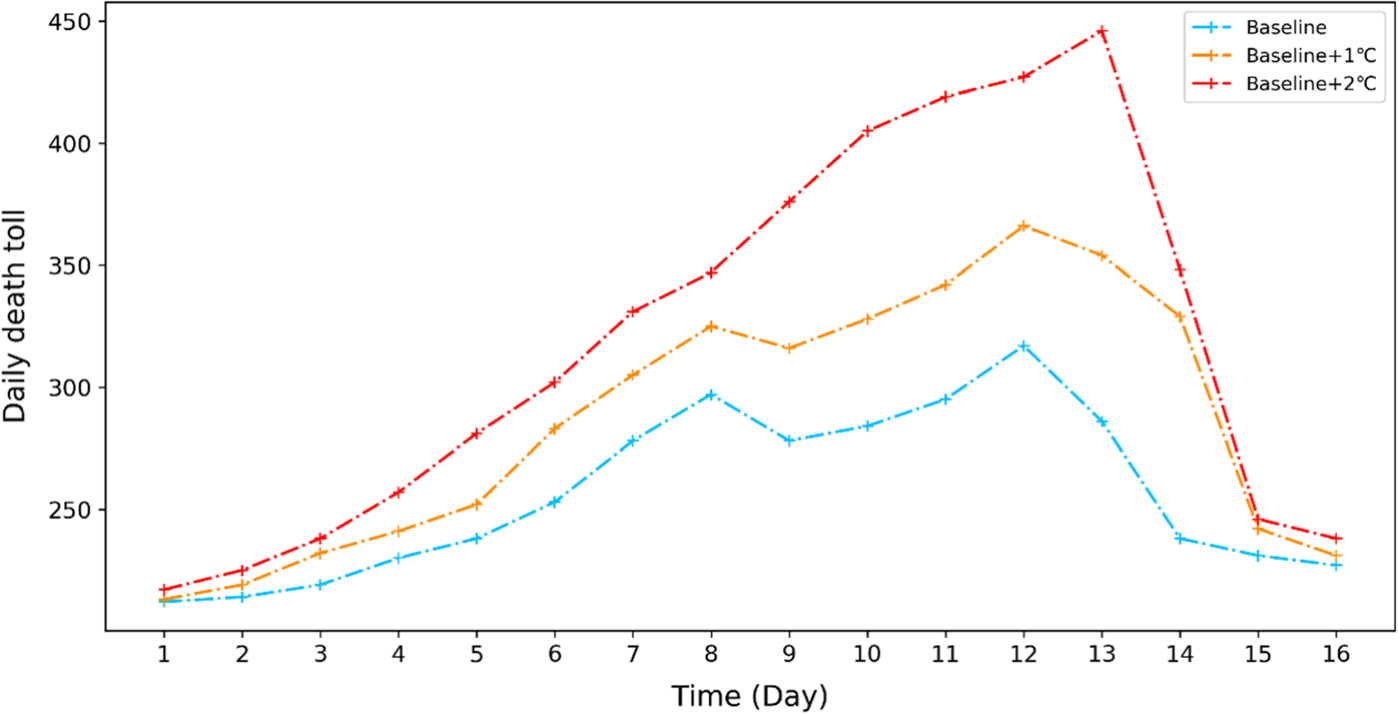
Simulations of the marginal impact of temperature increase on daily death tolls

The application of the UFCS-SD model for impact assessment and mitigation measure evaluations was carried out for the second longest heat wave event between 1960 and 2013, which lasted for 15 days in July 2013. The reference setting for this assessment purpose is the simulated death tolls of this heat wave event, called baseline. We then add 1 °C and 2 °C to the daily maximum temperature of the baseline and name these two testing scenarios as Baseline 1 and Baseline 2, respectively. Figure 6 presents the simulation results of the baseline and Baseline 1 and Baseline 2. It shows the increases in the daily death toll by an average of 11% and 23% in comparison with the baseline under Baseline 1 and Baseline 2, respectively. The increasing marginal effects imply significant challenges to Shanghai in dealing with heat wave shocks in the future because the maximum daily temperature is highly likely to increase with the trend of global warming.

### Evaluation of mitigation measures

**Fig. 7:**
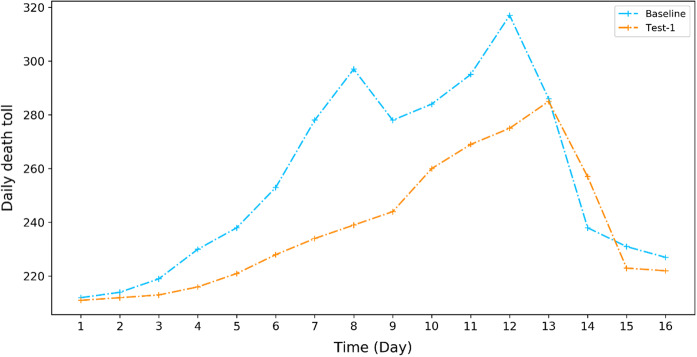
The simulated effect of hospital beds readiness (20%) on the number of daily deaths

Two types of mitigation measures are evaluated in this project. One is a short-term measure, referring to that the city’s health system makes 20% of total hospital bed available/ready for dealing with a heat wave shock once receiving the forecasting information. The choice of 20% is based on experts’ assessment of the heat wave events in July 2013. The simulation result as shown in Fig. 7 indicates that if the hospital could prepare 20% of their beds for emergency response to this long-lasting and extreme heat wave in July 2013, the most significant reduction of daily death toll could be achieved on days 7 and 8, being 44 (15.8%) and 58 (19.5%) in comparison with the baseline scenario where such a mitigation measure is not available.

**Fig. 8:**
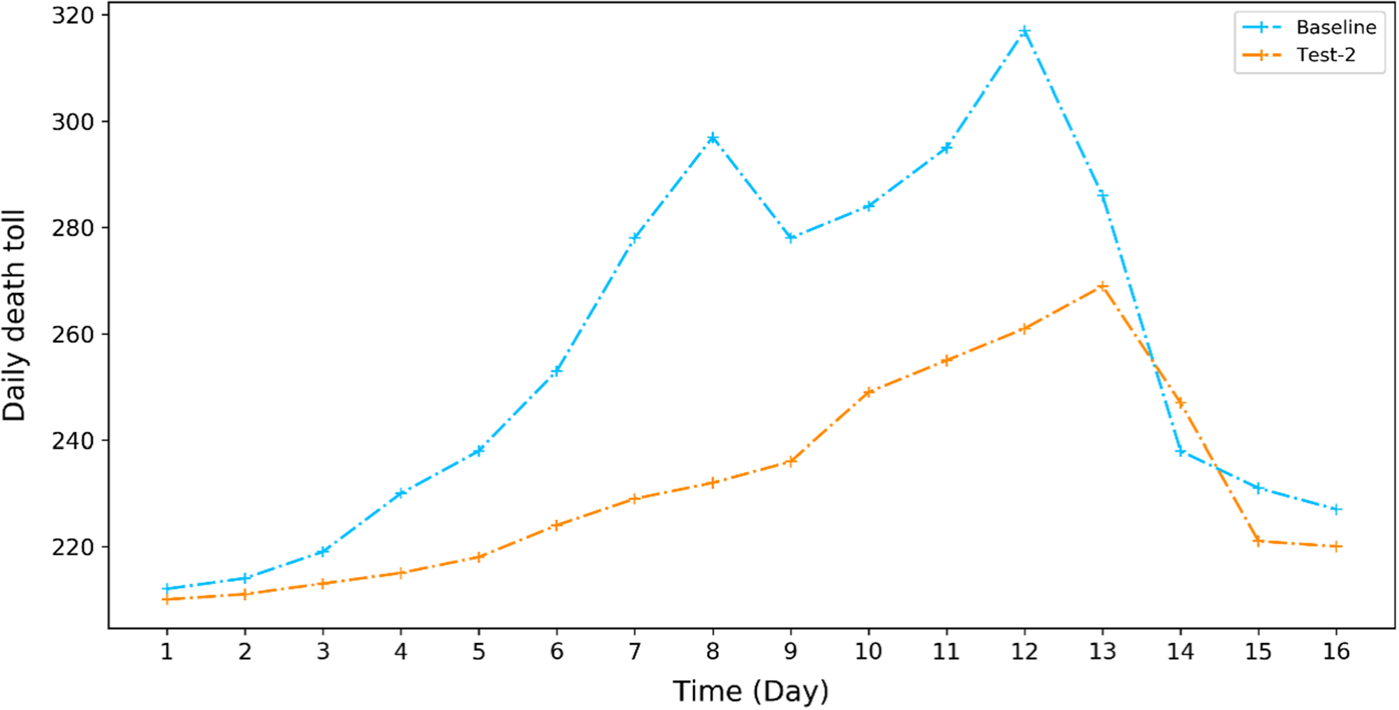
The simulated effect of increasing minimum living allowance by 20% on the number of daily deaths

The second mitigation measure is long-term-oriented and thus can be delinked with a specific forecasting of a heat wave event, but it is informed by the increasing marginal effects of 1 °C addition to a heat wave as discussed in "Marginal impact of 1 °C increase on daily death toll in July 2013" section above. This measure targets at the poorest residents in the city and grant them an increment in their minimum living allowance by 20% (from RMB 790/month to RMB 948/month). The simulation result as shown in Fig. 8 indicates that the most significant reduction of daily death toll could be achieved on days 8 and 12, being 65 (21.9%) and 56 (17.7%), respectively, in comparison with the baseline scenario where such a mitigation measure is not available.

## Summaries and discussion

A modern city is a complex system of sub-systems and is becoming increasingly vulnerable to extreme weather and climate events in the future. A weather/climate shock to any one of the subsystems may bring significant risk to the entire urban system if climate service is inactive and prevention/mitigation measures are weak. To conceptualize a systems analysis perspective in assessing the performance of potential mitigation measures in effectively reducing the climate anomaly induced human health risks, this paper has first presented a systems analysis version of urban framework for climate service (UFCS) in the context of Shanghai, a modern mega-city. It has employed this framework as a conceptual guiding to calibrate and verify a system dynamics (SD) modeling in the context of heat-related mortality in Shanghai and then applied this UFCS-SD model to evaluate the performances of two mitigation measures. The UFCS-SD model consists of five modules: climate module, social module, economic module, health module, and governance module. It considers 28 indexes including meteorological, population, social, economic, governance, and resilience indexes. The function relationships between variables are established and verified, and the model is verified using historical data to ensure its credibility.

The simulation result on the impact of heat wave intensity based on a real heat wave event in July 2013 shows an increasing marginal effect, meaning that when the heat wave intensity increased by 1 °C and 2 °C, respectively, the total number of daily deaths increased by 11% and 23%. Simulation results of a short-term mitigation measure show that if the hospital system could prepare 20% of beds available for emergency response to heat waves once receiving the warning in advance, the number of daily deaths could be reduced by 40–60 (15.8–19.5%) on the 2 days of day 7 and day 8, in comparison with the baseline scenario where such a mitigation measure is not available. Simulation results of a long-term mitigation measure show that if increasing the minimum living allowance of 790 RMB/month in 2013 by 20%, the number of daily deaths could be reduced by 50–70 (17.7–21.9%) on the 2 days of day 8 and day 12, in comparison with the baseline scenario where such a mitigation measure is not available.

An equally attractive, if not more, application of our SD model for evaluating mitigation options against climate hazards should be to the relationship between heat wave and heatstroke. However, such an application is constrained by data availability. Once the number of heatstroke cases per day becomes available, the above model can be easily extended to evaluate the performance of additional mitigation measures in reducing the occurrence of heatstroke cases. The tool developed and its application demonstrated in this research can help policy makers to systematically evaluate adaptation and mitigation options based on the quantified performance of those options, thus strengthening urban resilience to changing climate.

Some direct policy implications can be drawn from our research. First, it is fundamentally important to increase the accuracy of heat wave forecasting by enhancing scientific and technical progresses in the meteorological forecasting field. Second, given the increasing accuracy of existing forecasting, it is important for the health sector to have the prompt and effective responsibility. Such ability can significantly reduce mortality and human health risk when facing an extreme heat wave. Third, to make cooling equipment affordable by low-income households will greatly reduce the deadly consequences of an extreme heat wave event.
